# Colloidal-quantum-dot nanolaser oscillating at a bound-state-in-the-continuum with planar surface topography for a high *Q*-factor

**DOI:** 10.1515/nanoph-2024-0730

**Published:** 2025-03-31

**Authors:** Tae-Yun Lee, Hansol Lee, Heonsu Jeon

**Affiliations:** Department of Physics and Astronomy, 26725 Seoul National University, Seoul 08826, Republic of Korea; Inter-University Semiconductor Research Center, 26725 Seoul National University, Seoul 08826, Republic of Korea; Institute of Applied Physics, 26725 Seoul National University, Seoul 08826, Republic of Korea

**Keywords:** nanolaser, colloidal quantum dots, photonic crystal, bound state in the continuum, squeegee sweeping

## Abstract

Solution-based optical gain materials offer a cost-effective path to coherent light sources. Further, bound states in the continuum (BICs) have garnered great interest owing to their diverging quality (*Q*) factors. Therefore, a hybrid of these – a solution-based material for optical gain and a BIC structure for the lasing mode – should constitute an ideal form factor for low-cost and low-threshold nanolasers. However, the nonuniform surface topography induced during the thin-film formation of a solution-based material, especially on top of a prepatterned substrate, can easily disrupt the structural symmetry required for a high-*Q* BIC, resulting in a degradation of *Q*. Thus, in this study, a simple surface-flattening technique utilizing a soft and flexible squeegee was applied, which realized the planar surface topography crucial for preserving the high *Q* promised by the BIC and achieving low-threshold lasing. We fabricated BIC nanolasers by incorporating colloidal quantum dots (CQDs) for optical gain into a two-dimensional photonic crystal backbone layer composed of Si_3_N_4_. By leveraging the unique properties of the BIC mode with a well-ordered surface, our CQD-based BIC laser exhibited a lasing threshold as low as 10.5 kW/cm^2^, which is significantly lower than those reported in previous studies.

## 1 Introduction

The advent of microdisk lasers [1], [2], [3] triggered tremendous interest in lasers with micro- or nano-scale footprints because of their potential use as compact coherent light sources that can be used in numerous applications, such as high-density photonic integrated circuits [4], [5], [6], bioimaging [7], [8], biosensors [9], [10], [11], spectrometer [12], [13], and display [14]. Since then, a variety of small-scale laser platforms have been developed, including microdisks [1], [2], [3], photonic crystals (PhCs) [15], [16], [17], nanowires [18], [19], and localized surface plasmons [20], [21], [22]. However, for practical use in the future, such small-scale lasers must simultaneously be prepared at a low cost and exhibit low thresholds. Concerning optical gain materials for lasing, solution-based systems – represented by inorganic colloidal quantum dots (CQDs) [23] and hybrid perovskite nanocrystals [24] – are advantageous because they can be chemically synthesized and easily configured into thin films by a simple spin-coating process, in contrast to epitaxially grown semiconductor quantum-well heterostructures. In particular, CQDs are of great interest because they possess additional unique advantages such as broad and high absorption bands [25], color tunability according to nanoparticle size [26], high internal quantum efficiencies (> 90 %) [27], [28], [29], [30], and rich material systems available for the visible to near-infrared range [31].

However, for low-threshold lasing, a cavity structure that can offer a high quality factor (*Q*) is required. This can be satisfactorily fulfilled by *bound states in the continuum* (BICs). Although BICs were originally predicted in quantum-mechanical systems [32], they have a greater impact and have thus been adopted in the field of photonics, owing to their unusual means of confining light without any radiation into free space, and thus an infinite *Q* [33], [34]. Among the various types of BICs, symmetry-protected BICs can appear in periodic structures such as two-dimensional (2D) PhCs [35]. A Γ-point eigenmode that has even symmetry under 180° rotation (*C*_2_) is decoupled from radiating plane waves that are odd under *C*_2_, thus qualifying as a symmetry-protected BIC. However, structural imperfections can easily break *C*_2_ symmetry and degrade the infinite *Q* of the BIC structure, rendering it finite. When a laser based on a BIC is prepared from a solution-based material, it is particularly susceptible to structural imperfections. For example, spin coating leads to a gradation in film thickness because of the radially varying centrifugal force, and to a partially conformal (and therefore wavy) surface topography when spun on a prestructured substrate [36].

In this study, a symmetry-protected PhC BIC laser was developed, in which a square lattice array of cylindrical air holes etched into a high-refractive-index dielectric slab and subsequently filled with CQDs was used for optical gain. To preserve the high *Q* of the resultant BIC structure, a smooth and planar surface topography across the entire device is critical. To address this challenge, a soft and flexible *squeegee* was used to remove excess CQDs from the device surface, resulting in a planar device surface with CQDs filling the air holes fully, thereby producing a high *Q* and low lasing threshold.

## 2 Results and discussion

### 2.1 Design of CQD-BIC laser structure

**Figure 1:**
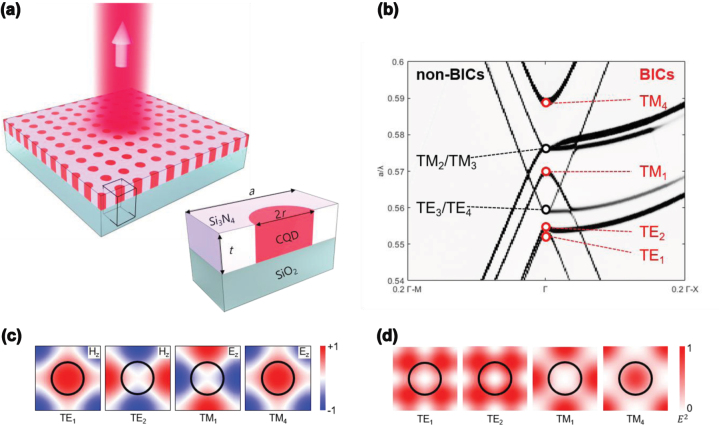
CQD-based 2D PhC BIC laser structure. (a) Schematic of the laser structure comprising a Si_3_N_4_ backbone layer with a square-lattice array of air holes filled with CQDs. The inset shows a cross-section of the PhC unit cell. (b) Photonic band structure calculated for the 2D PhC shown in (a). The Γ-point band-edge modes are categorized into BICs (hollow red circles) and non-BICs (hollow black circles). (c) Field profiles of the four Γ-point BIC modes: *H*_z_ for TE_1_ and TE_2_ and *E*_z_ for TM_1_ and TM_4_. (d) Normalized modal intensities $E^{2}=E_{x}^{2}+E_{y}^{2}+E_{z}^{2}$ of the four Γ-point BIC modes. Each profile in (c) and (d) is shown across a unit cell, in which the black circle represents the air-hole boundary.

Figure 1(a) shows the proposed CQD-based BIC laser structure, comprising a Si_3_N_4_ slab containing a 2D square-lattice PhC array of air holes filled with CQDs over a silica substrate. The Si_3_N_4_ (*n*_H_ = 2.02) and dense CQD aggregates (*n*_L_ = 1.75) constitute the high- and low-refractive-index materials of the resultant 2D PhC structure, respectively. The refractive index contrast (Δ*n* = 0.27) is too small for a full bandgap but large enough to induce partial bandgaps and associated band edges at the Γ-point (*k*_||_ = 0) (Figure 1(b)). Under optical excitation, the dense CQD aggregates inside the air holes provide an optical gain at approximately *λ* = 620 nm. The Si_3_N_4_ backbone layer is 260-nm thick; this ensures that it supports only two fundamental waveguide modes at the CQD emission wavelength *λ* ≈ 620 nm: one in transverse-electric (TE) and the other in transverse-magnetic (TM) polarization.

Finite-difference time-domain (FDTD) simulations were conducted to obtain the photonic band structure, as shown in Figure 1(b). Eight Γ-point band-edge modes were identified in total, including two pairs of degenerate modes, labelled TE_1_–TE_4_ and TM_1_–TM_4_. Only four Γ-point band-edge modes (TE_1_, TE_2_, TM_1_, and TM_4_) possessed an even modal symmetry – the requirement for symmetry-protected BICs. Their field profiles, *H*_z_ for the TE modes and *E*_z_ for the TM modes, are shown in Figure 1(c). The other band-edge modes (TE_3_/TE_4_ and TM_2_/TM_3_) had odd symmetries (Supplementary Information S1); thus, they were discounted as BICs. The extreme *Q* values of the BIC band-edge modes were confirmed by two additional simulations: the modal couplings of incoming plane waves into the PhC structure (Supplementary Information S2) and the modal decays out of the PhC structure (Supplementary Information S3). These results agreed perfectly with the BIC identifications based on modal symmetry.

However, a low-threshold laser requires not only a high-*Q* cavity mode but also a strong spatial overlap between the cavity mode and the optical gain material (CQDs in the present case). Although the BIC mode guarantees a high *Q* value, the condition for modal overlap must be addressed separately. Figure 1(d) shows the electric field intensity profiles ( $E^{2}=E_{x}^{2}+E_{y}^{2}+E_{z}^{2}$ ) calculated for the four Γ-point BIC modes. Modal overlap was considered regardless of the direction of the electric field because the orientations of the electric dipoles inside the CQDs are presumably random. TM_4_ clearly exhibited the highest electric field overlap with the CQDs located inside the air holes; the calculated overlap factors were 0.12, 0.16, 0.04, and 0.31 for TE_1_, TE_2_, TM_1_, and TM_4_, respectively. The physical dimensions of the PhC structure were then determined such that the TM_4_ mode matched the CQD emission wavelength *λ* = 620 nm, which resulted in *a* = 365 nm for *r*/*a* = 0.25, where *a* and *r* are the PhC lattice constant and air-hole radius, respectively.

### 2.2 Effects of structural imperfections

**Figure 2:**
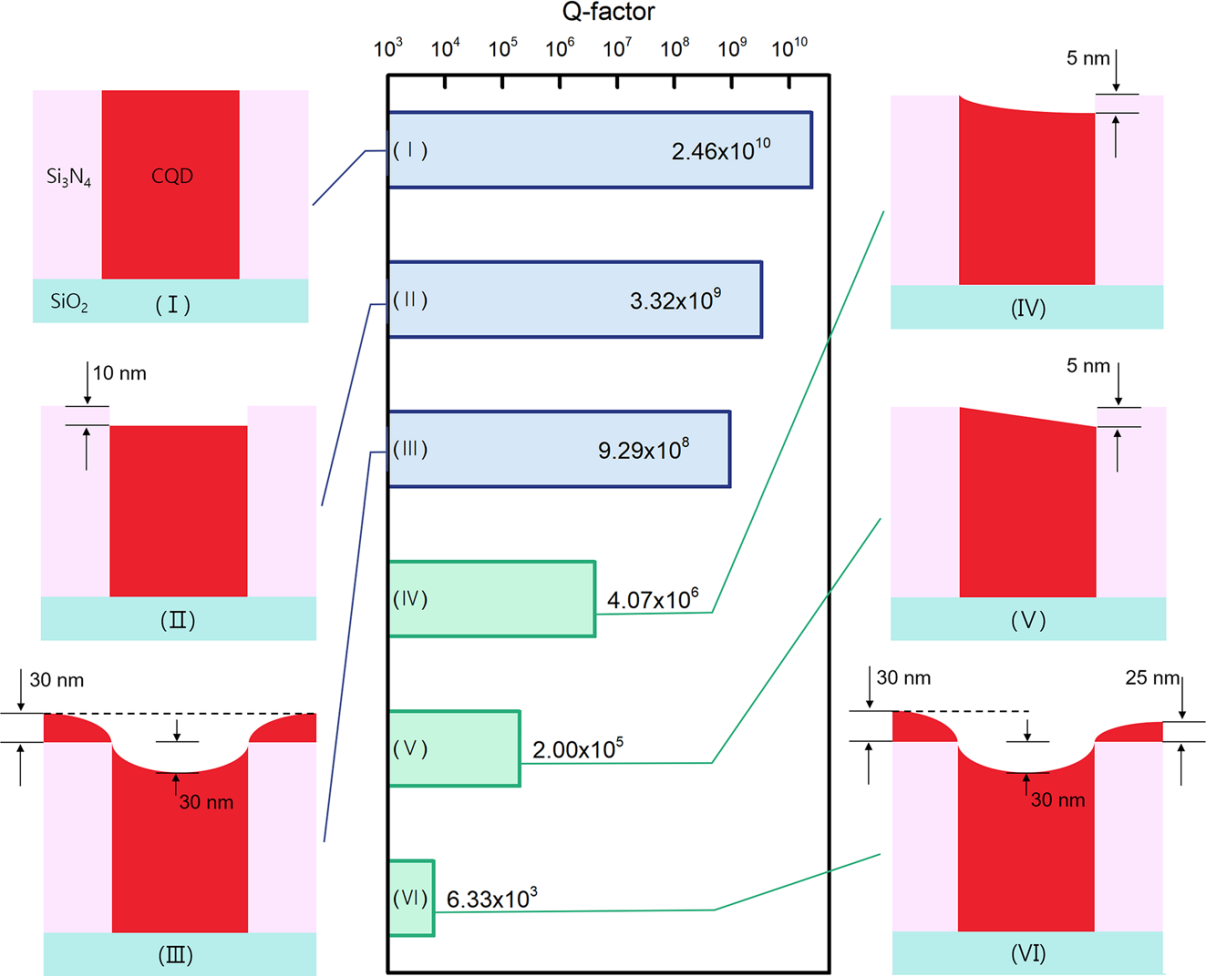
*Q*-factors of the TM_4_ Γ-point BIC mode calculated for various CQD profiles around the air holes. Each bar of the graph is presented with a schematic of the device cross-section showing the CQD surface profile – drawn with exaggeration: (I) ideal, (II) indented with a flat surface, (III) partially conformal and symmetric, (IV) slanted with a curved surface, (V) slanted with linear slope, and (VI) partially conformal but asymmetric. Note that (I)–(III) are symmetric whereas (IV)–(VI) are asymmetric. The thickness dimensions used in the model calculations are specified.

Although a BIC theoretically offers infinite *Q* and zero loss, any structural imperfection can degrade *Q* to a finite value because it may disrupt the conditions required for symmetry-protected BICs. To examine the effects of imperfections, FDTD simulations were performed for various device surface topographies; however, to facilitate the simulations, model structures were simplified to one-dimensional gratings rather than 2D square-lattice PhCs, in which there is only one BIC and one non-BIC for both TE and TM polarizations. The simulation results for the BIC TM mode, corresponding to TM_4_ in the 2D PhC counterpart, are summarized in Figure 2. An ideal device with a planar surface (I) exhibits an extremely high *Q* value (*Q* ≈ 10^10^). When structural perfection is lost, either by a small CQD indent into the air holes (II) or by a partially conformal CQD deposition (III), the *Q* value marginally reduces but remains high (*Q* ≈ 10^9^). We attribute the small reduction in *Q* to the fact that the *C*_2_ symmetry itself is retained. In contrast, the *Q* value dramatically decreases when the *C*_2_ symmetry is broken: *Q* ≈ 10^5^–10^6^ for curved (IV) or linear (V) slanted CQD profiles and *Q* ≈ 10^3^–10^4^ for a partially conformal and asymmetric CQD profile (VI). Therefore, preservation of *C*_2_ symmetry during device fabrication is crucial. For comparison, we performed similar simulations for the non-BIC TM mode (Supplementary Information S4), which corresponds to TM_2_/TM_3_ in the 2D counterpart. The results indicate that the sensitivity of *Q* to imperfections was far lower for the non-BIC mode than for the BIC mode.

The non-ideal CQD profiles (II–VI) do not originate from imagination but reflect plausible outcomes when the CQDs are spin-coated. Spin coating has been successfully used to construct thin and uniform CQD films on flat surfaces [37]; however, it results in a wavy surface when applied to a patterned substrate [38], [39]. In this context, Profiles II and III may be obtained when dilute and dense CQD solutions are spin coated, respectively, whereas Profiles IV/V and VI are the counterparts of II and III when spin coating is performed off-center so that a substantial amount of centrifugal force is exerted in the radial direction. Therefore, spin coating is not a desirable process for depositing CQDs for high-quality BIC lasers because it is difficult to produce planar and symmetric surface topography on a PhC backbone structure. A simple and straightforward solution is *squeegee sweeping*, which is inspired by an automobile windshield wiper that mechanically removes dirt from the surface. Using this method, excess CQDs on the surface of a prepatterned PhC backbone structure can be effectively removed, while the CQDs inside the air holes remain intact. In fact, the method was successfully used to insert biological entities or metallic nanoparticles into wells or slots of micrometer or nanoscale dimensions [40], [41]. We also applied this method previously to the fabrication of PhC phosphors and improved their performance [42], and now to the fabrication of BIC lasers with flat surface and thus high *Q*.

### 2.3 Fabrications

**Figure 3:**
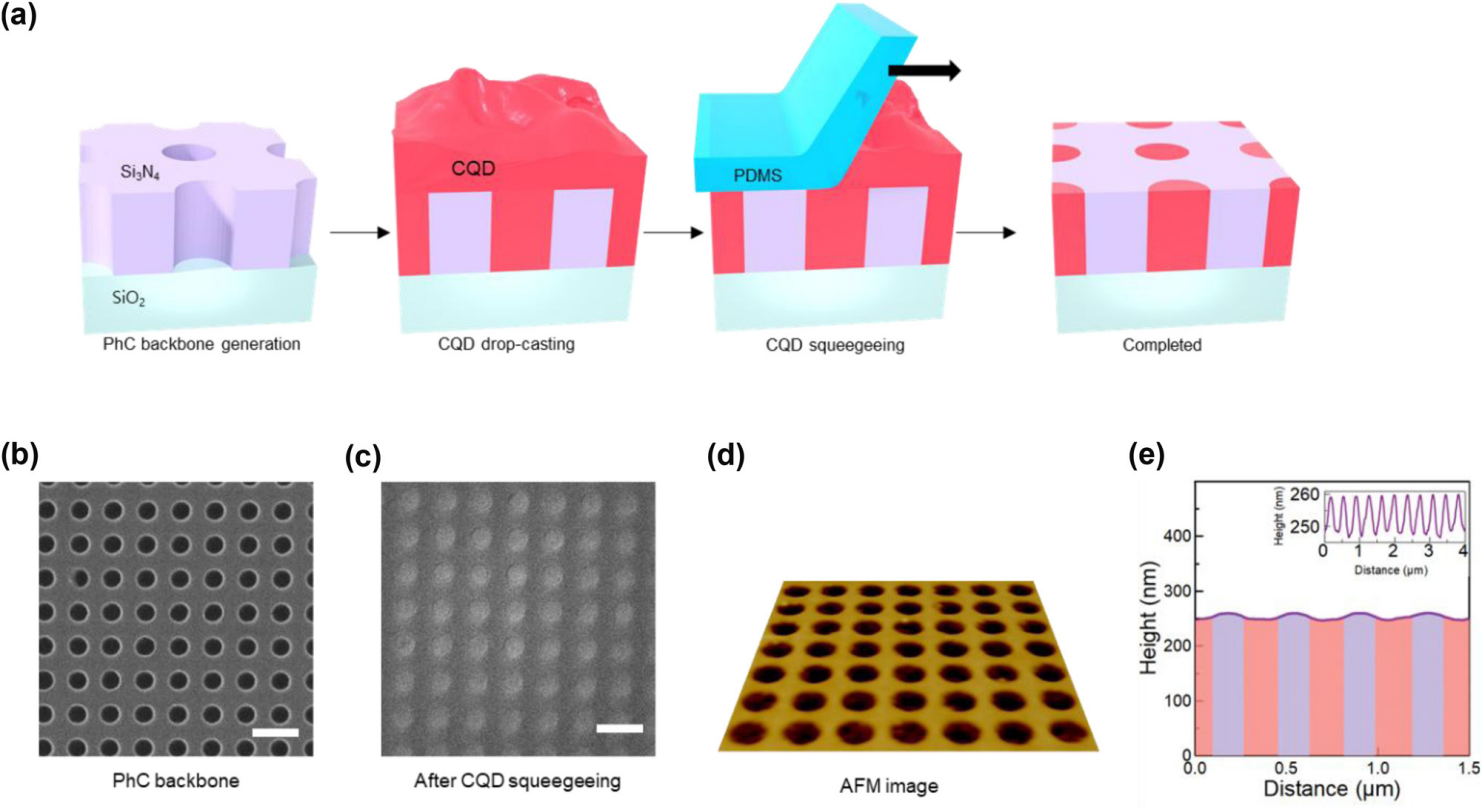
Fabrication of the surface-planarized CQD-based BIC laser device. (a) Fabrication steps for the BIC laser with a planar surface. (b) Top-down SEM image of the Si_3_N_4_ PhC backbone slab with a square-lattice array of empty air holes (before CQD deposition). (c) Top-down SEM image of a completed device with the air holes filled selectively with dense CdSe/ZnS CQDs. The scale bars are 500 nm. (d) Perspective AFM image of a completed device. (e) Height profile across a line that bisects a series of the CQD-filled air holes.

Figure 3(a) illustrates the key steps involved in the fabrication of the CQD-BIC laser device. First, a 260-nm-thick Si_3_N_4_ layer was deposited by low-pressure chemical vapor deposition on top of a 1-μm-thick SiO_2_ grown on silicon substrate, followed by the deposition of a 10-nm-thick Cr layer via e-gun evaporation. The Si_3_N_4_ layer is a dielectric slab from which the 2D PhC backbone structure was formed, whereas the Cr layer serves as a hard mask during Si_3_N_4_ etching. Using electron beam lithography, a 2D PhC pattern composed of a square lattice array of air holes (lattice constant *a* = 365 nm, hole radius *r*/*a* = 0.25) was generated over an area of 300 μm × 300 μm. The PhC pattern was then transferred sequentially to the underlying Cr and Si_3_N_4_ layers via reactive-ion etching. Chemical removal of the remaining Cr layer completed the fabrication of the PhC backbone structure. Figure 3(b) shows a top-down scanning electron microscopy (SEM) image that demonstrates the structural quality and dimensions of the 2D PhC backbone structure etched into the Si_3_N_4_ layer.

Afterwards, a small amount of CQD solution (CdSe/ZnS core–shell CQDs dispersed in toluene) was drop-cast onto the Si_3_N_4_ PhC backbone. Once the toluene evaporated naturally, acetone was sprayed onto the sample, immediately followed by squeegee sweeping to remove excess CQDs from the sample surface. The squeegee was prepared by cutting a piece of polydimethylsiloxane (PDMS) with a sharp razor blade. The acetone played the dual roles of softening the CQD aggregate and lubricating the squeegee sweeping. The SEM image in Figure 3(c), which was captured after squeegee sweeping, clearly demonstrates that the excess CQDs were thoroughly removed from the device surface, whereas the air holes remained filled with CQDs without voids. Atomic force microscopy (AFM) was used to examine the overall degree of surface planarization. An AFM image is shown in Figure 3(d), while the surface profile along a line across multiple air holes is plotted in Figure 3(e). The air holes were slightly indented to a depth of approximately 10 nm. As mentioned previously, this type of small height variation does not excessively deteriorate *Q* as long as the surface profile remains symmetric. However, squeegee sweeping occurs in a unidirectional manner so that the actual CQD profile is expected to be somewhere between Profiles II and IV/V in Figure 2. Resultant PhC structure should still be able to offer a reasonably high *Q* for low-threshold lasing action.

### 2.4 Measurements

**Figure 4:**
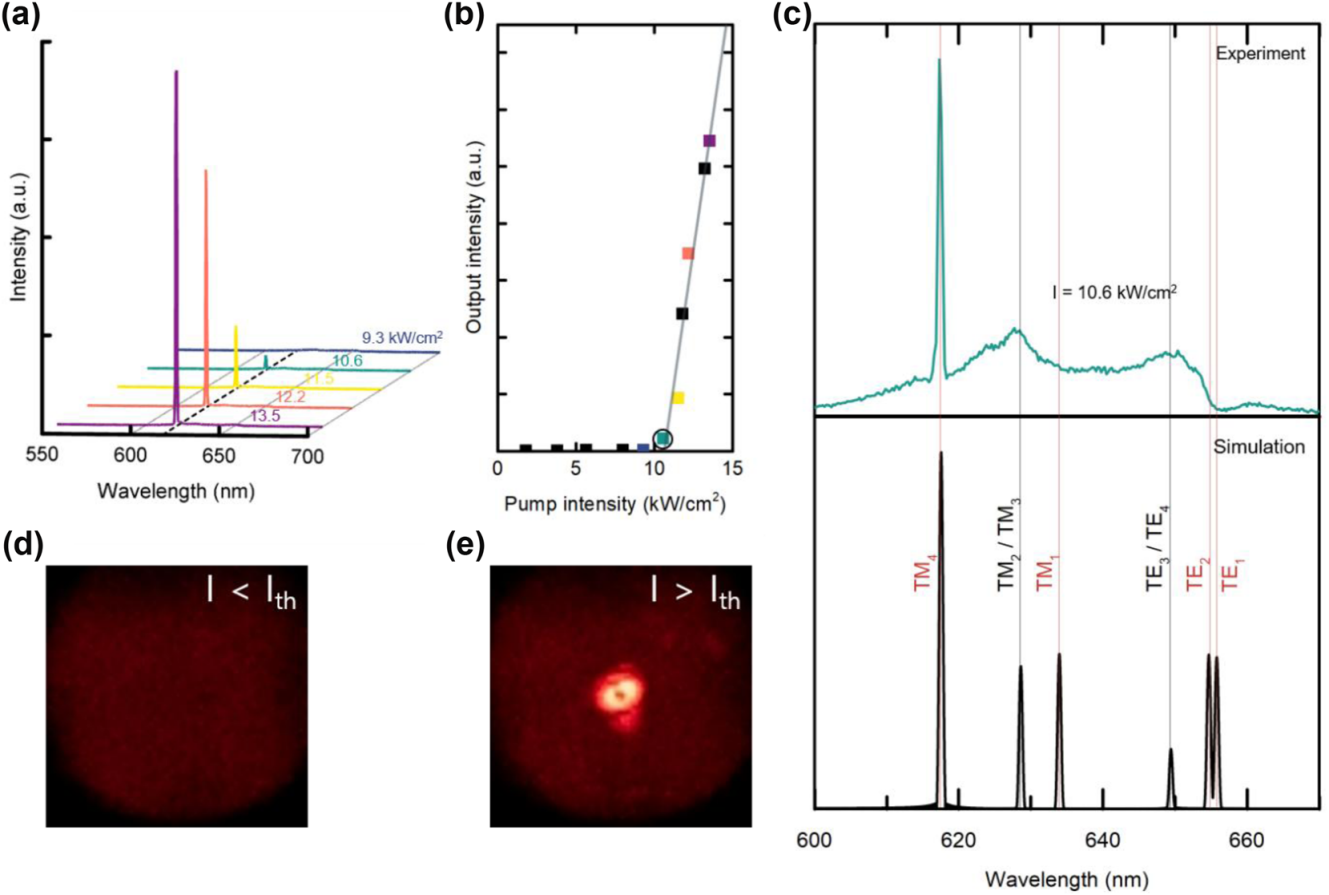
Performance characteristics of the CQD-BIC laser. (a) Emission spectra when excited at various pump intensities: *I* = 9.3, 10.6, 11.5, 12.2, and 13.5 kW/cm^2^. (b) Light-in versus light-out curve. From the plot, the lasing threshold was determined as *I*_th_ ≈ 10.5 kW/cm^2^. (c) Emission spectrum recorded near threshold at *I* = 10.6 kW/cm^2^ (upper) and FDTD-simulated spectrum (lower). Far-field emission patterns imaged when excited (d) below and (e) above the threshold.

The fabricated CQD-BIC laser device was optically excited using a frequency-doubled 532-nm Nd:YAG laser in pulsed mode (pulse width = 500 ps, repetition rate = 1 kHz). A 5× objective lens was used to focus the pump laser beam onto the device in a spot of approximately *ϕ* = 260 μm (full-width half-maximum) in diameter. Figure 4(a) shows the CQD emission spectra recorded at several representative excitation levels, exhibiting sharp single-mode lasing peaks above a certain excitation level. The light-in versus light-out (*L*−*L*) relationship is plotted in Figure 4(b) to determine the threshold. From the *x*-intercept of a linear fit to the data points, an approximate threshold excitation intensity of *I*_th_ = 10.5 kW/cm^2^ was obtained. By comparing this value with those obtained from our previous CQD-based PhC lasers [43], [44], [45], [46], we found that the threshold of the present CQD-BIC laser was reduced by a factor of 10–100 (Supplementary Information S5). In huge contrast, spin-coated device without squeegee sweeping exhibited a lasing threshold 10 times higher than that of the squeegee-swept CQD-BIC laser, showing the negative impact of nonuniform surface topography induced during the spin-coating process. Even worse, drop-cast device without squeegee sweeping showed no lasing at all, lacking discernible resonant modes due to excessive CQD accumulation (Supplementary Information S6).

The upper panel of Figure 4(c) shows the experimental spectrum recorded at an excitation power level near the threshold (*I* = 10.6 kW/cm^2^). In the figure, three optical modes can be identified, including one that evolves into a lasing peak at approximately 620 nm. FDTD simulations were performed to theoretically confirm the modes. The results are shown in the bottom panel of Figure 4(c), where all the Γ-point band-edge modes manifested; from the shortest to longest wavelength: TM_4_, TM_2_/TM_3_, TM_1_, TE_3_/TE_4_, TE_2_, and TE_1_. Reflecting the experimental margins, the structural parameters of the simulated PhC model were fine-tuned to obtain the best agreement with the experimental spectrum. A comparison between the experimental data and simulation results indicates that the three experimentally identified modes correspond to one BIC (TM_4_) and four degenerate non-BICs (TM_2_/TM_3_ and TE_3_/TE_4_), and the lasing itself occurs at TM_4_, the BIC mode that was aimed at and expected for lasing. The absence of the other BIC modes – TM_1_, TE_1_, and TE_2_ – in the experimental spectrum can be attributed to the fact that they belong to *dielectric bands* so that their electric field maxima coincide with the high-refractive-index region (Si_3_N_4_) and thus have little overlap with the CQDs inside the air holes, as mentioned in conjunction with Figure 1(d).

Figure 4(d) and (e) show photographs of the laser emission captured below and above the laser threshold, respectively. A long-pass filter with a cutoff wavelength of 575 nm was placed in front of the camera to exclude scattered pump laser light and thus capture only the red CQD emission. Below the threshold, only a weak and wide emission pattern was observed, which is characteristic of spontaneous emission. Above the threshold, an intense emission spot was clearly visible. Interestingly, the laser spot above the threshold was donut shaped; its center was dark. However, this is a well-known property of a Γ-point band-edge laser [47], and thus, more evidence that the Γ-point band-edge mode is responsible for CQD lasing.

## 3 Conclusions

To realize a low-cost, low-threshold nanolaser, a hybrid nanolaser device was designed and fabricated by combining a square-lattice 2D PhC backbone slab (for a high-*Q* BIC mode) and CQDs (for cost-effective optical gain). Although spin coating is a convenient method for preparing thin CQD films, it inherently results in nonuniformities when spun on a prepatterned PhC backbone structure. Simulations were conducted to investigate the adverse effects of structural non-uniformities, indicating that a planar surface topography is critically important for preserving the high *Q* of the BIC mode. A novel process termed *squeegee sweeping* was applied to remove excess CQDs from the surface and simultaneously fill the air holes with CQDs. The resultant device exhibited an overall planar surface, which in turn reduced the unevenness that can cause radiation loss. The fabricated devices lased in single-mode at a threshold excitation intensity of approximately 10.5 kW/cm^2^, which is the lowest value reported thus far.

## 4 Methods

### 4.1 FDTD simulations

All numerical simulations were performed using a commercial software package (Lumerical FDTD, Ansys Inc.) based on the FDTD method. The complex refractive indices of dense Si_3_N_4_ and SiO_2_ films were obtained from the work by Philipp [48] and the handbook by Palik [49], respectively. Spectroscopic ellipsometry was used to determine the complex refractive indices of the CQD films (Supplementary Information S7).

### 4.2 Device fabrication

A 1-µm-thick oxide layer was grown on a p-type silicon wafer through wet oxidation at 900 °C in a furnace (SHF-150, Seltron). A 260-nm-thick Si_3_N_4_ film was deposited using low-pressure chemical vapor deposition (SHF-150-L, Seltron) at 785 °C, followed by the deposition of a 10-nm-thick Cr layer using e-gun evaporation. An e-beam resist (ZEP520A, Zeon) was spin coated onto the cleaned Cr layer at 4,000 rpm, resulting in a thickness of 300 nm. To reduce the charging effects during e-beam lithography, a charge-dissipating agent (ESPACER 300Z, RESONAC) was coated on top of the e-beam resist. E-beam lithography (JBX-6300FS, JEOL) was employed to produce PhC patterns with an accelerating voltage 100 keV and a current of 1 nA. The PhC pattern in the e-beam resist was subsequently transferred to the underlying Cr and Si_3_N_4_ layers via reactive-ion etching (Plasmalab 80 Plus, Oxford Instruments) using a Cl_2_/O_2_ gas mixture (20:20 sccm) for Cr and a CF_4_/O_2_/N_2_ gas mixture (40:5:5 sccm) for Si_3_N_4_, respectively. Chemically synthesized CdSe-ZnS core-shell red CQDs (CZO-620T, Zeus) dispersed in toluene solvent at 0.6 wt% were drop casted and swept using a PDMS squeegee to remove excess CQDs from the surface. To prepare a PDMS block, a commercial silicone elastomer kit (SYLGARD, Dow) was used with a base-to-curing agent weight ratio of 7:1. The mixture was degassed in a desiccator for 10 min to remove air bubbles and subsequently cured at room temperature for 2 days. The cured PDMS was then cut into approximately 10 mm × 30 mm blocks with a thickness of approximately 3 mm for convenience (Supplementary Information S8). After spraying acetone onto the drop-casted CQD sample as a lubricant, the sweeping was performed manually at approximately 1 mm/s in alternating left-to-right and right-to-left motions to minimize directional bias in the results.

### 4.3 SEM and AFM measurements

High-resolution imaging of the surface morphology was performed using field-emission scanning electron microscopy (FE-SEM) (MIRA3, Tescan). For a more in-depth analysis of the surface topography, non-contact atomic force microscopy (AFM) (NX-10, Park Systems) was employed to acquire quantitative depth information. Measurements were performed using an AFM tip with the radius of curvature of ∼7 nm (PPP-NCHR, Park Systems). The raw AFM data were subsequently analyzed using XEI software (Park Systems) to extract the depth profile and 2D images.

### 4.4 Optical measurements

The fabricated devices were optically pumped using a frequency-doubled 532-nm Nd:YAG laser (PNP-M08010-130, Teem Photonics) in pulse mode (pulse width = 500 ps, repetition rate = 1 kHz). A 5× objective lens (NA = 0.13) was used to focus the pump laser beam to a spot approximately 260-μm in diameter. The emission spectra were measured vertically using a spectrometer (Kymera 193i-A Spectrometer with an iVac 316 CCD, ANDOR).

## Supplementary Material

Supplementary Material Details
