# Supplementary material for: Colloidal-quantum-dot nanolaser oscillating at a bound-state-in-the-continuum with planar surface topography for a high Q-factor
Source: Nanophotonics. 2025 Mar 31;14(10):1645–52. doi: 10.1515/nanoph-2024-0730 (PMC12116273; doi:10.1515/nanoph-2024-0730)
Supplement: Supplementary file 1 — Supplementary Material Details [file j_nanoph-2024-0730_suppl_001.docx]

[Supplementary Information]

Colloidal-quantum-dot nanolaser oscillating at a bound-state-in-the-continuum with planar surface topography for a high Q-factor

Tae-Yun Lee^#1,2^, Hansol Lee^#1,2^, and Heonsu Jeon^*1,2,3^

^1^Department of Physics and Astronomy, Seoul National University, Seoul 08826, Republic of Korea

^2^Inter-university Semiconductor Research Center, Seoul National University, Seoul 08826, Republic of Korea

^3^Institute of Applied Physics, Seoul National University, Seoul 08826, Republic of Korea

^#^Contributed equally

^*^Correspondence: Heonsu Jeon (hsjeon@snu.ac.kr)

List of Contents

[S1. Non-BIC nature of TE_3_/TE_4_ and TM_2_/TM_3_ band-edge modes 3](#_Toc190599252)

[S2. Modal couplings with incoming plane waves 4](#_Toc190599253)

[S3. Time evolution of the Γ-point band-edge modes 5](#_Toc190599254)

[S4. *Q*-factor of the non-BIC mode 6](#_Toc190599255)

[S5. Comparison of lasing thresholds for CQD-based lasers 7](#_Toc190599256)

[S6. Emission spectra from CQD-BIC laser structures prepared by drop casting and spin coating (without squeegee sweeping) 8](#_Toc190599257)

[S7. Complex refractive index dispersions of CQD film 9](#_Toc190599258)

[S8. Photographic and optical microscopy images of the PDMS squeegee 10](#_Toc190599259)

[S9. CQD surface qualities without squeegee sweeping 11](#_Toc190599260)

S1. Non-BIC nature of TE_3_/TE_4_ and TM_2_/TM_3_ band-edge modes


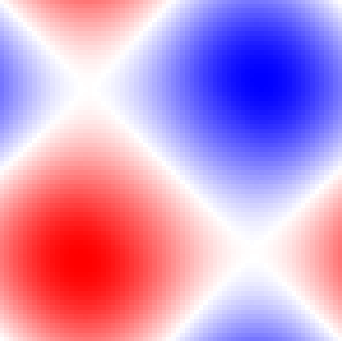

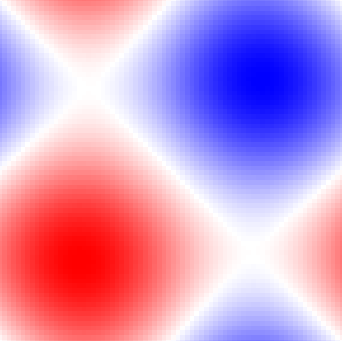


TE_3_ / TE_4_

TM_2_ / TM_3_


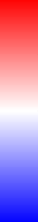


+1

-1

H_z_

E_z_

Fig. S1: Field strength profiles of the degenerate TE_3_/TE_4_ and TM_2_/TM_3_ Γ-point band-edge modes. (Left) *H*_z_ profile for TE_3_/TE_4_; (right) *E*_z_​ profile for TM_2_/TM_3_. The both modes exhibit odd parity under C_2_​ symmetry operation, implying that their radiation into free space is allowed.

S2. Modal couplings with incoming plane waves


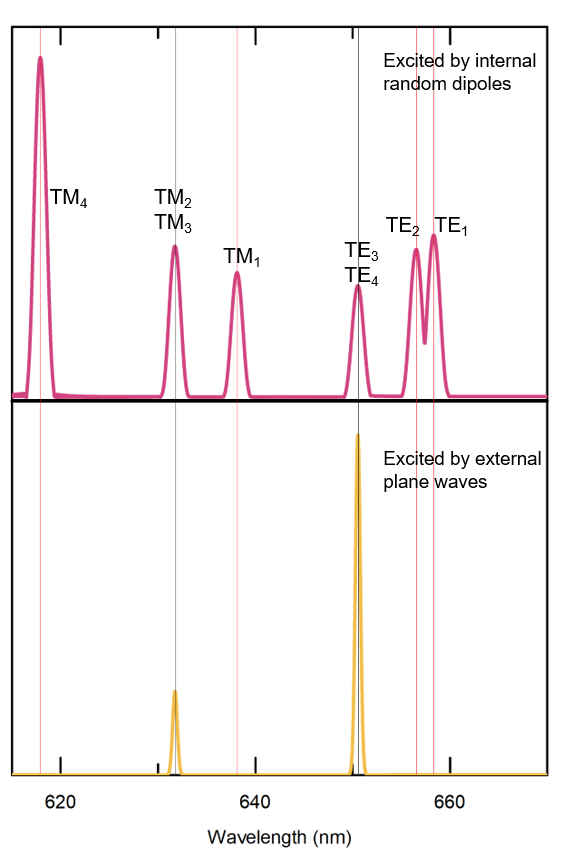


Fig. S2: FDTD simulation results on mode excitations. (Upper) Modal spectrum obtained with electric dipole sources located inside the PhC structure and distributed randomly, in which all the Γ-point band-edge modes are identified. (Lower) Modal spectrum obtained with external plane waves incident on the PhC structure, which reveals only the non-BIC modes as their low *Q*-factors (due to odd parity under C_2_ rotation) allow efficient couplings with the external plane waves.

S3. Time evolution of the Γ-point band-edge modes


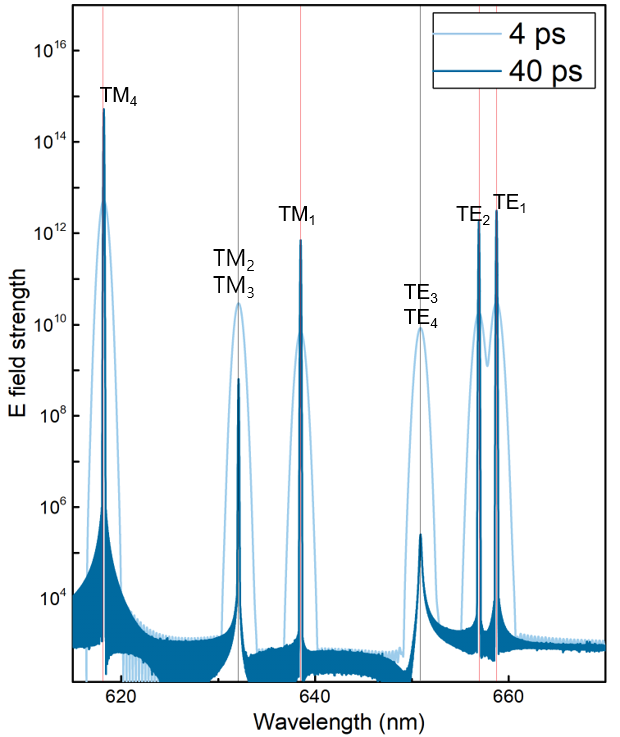


Fig. S3: Simulated time evolution of the Γ-point band-edge modes. Modal spectrum taken at 4 ps (light blue)⎯after a single oscillation of the electric dipoles distributed randomly inside the PhC structure⎯identifies all the Γ-point band-edge modes. After 40 ps (dark blue), the BIC modes (TE_1_, TE_2_, TM_1_, and TM_4_) manifest themselves even more vividly with enhanced electric field strengths. In good contrast, the electric field strengths of the non-BIC modes (TE_3_/TE_4_ and TM_2_/TM_3_) are lowered, indicating that the modes decay in time as they are allowed to couple to free-space modes.

S4. *Q*-factor of the non-BIC mode

**
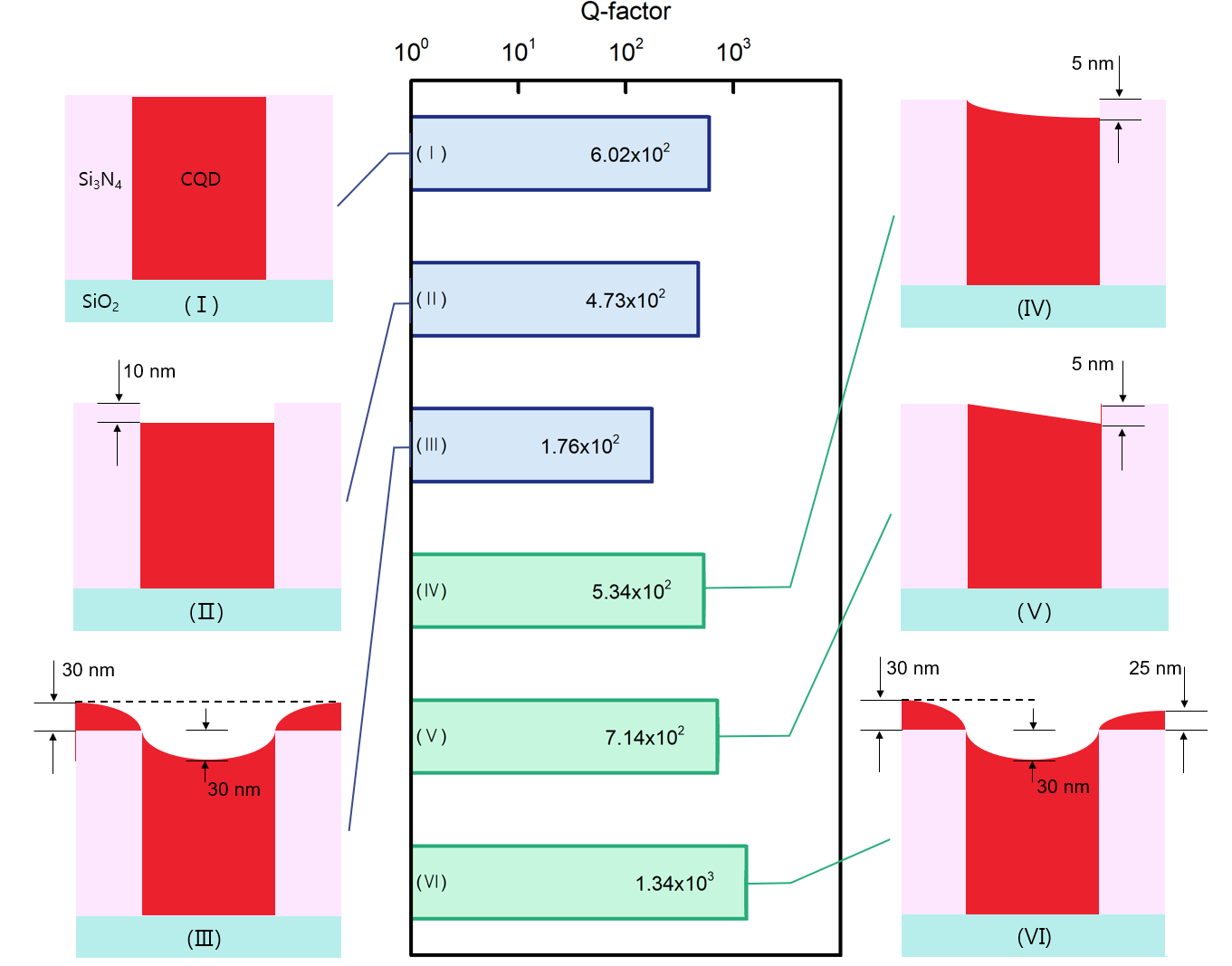
**

Fig. S4: *Q*-factors of the 1D non-BIC mode calculated for various CQD profiles. The non-BIC mode exhibit quite low *Q*-factors regardless of the CQD profiles, including the ideal planar surface topography in (I). This result is presumably due to the allowance of its coupling to radiation into free space and indicates the inferiority of the non-BIC mode for lasing.

S5. Comparison of lasing thresholds for CQD-based lasers

| **Article** | **Threshold** |
| --- | --- |
| H. Jung et al., "Tunable colloidal quantum dot distributed feedback lasers integrated on a continuously chirped surface grating", Nanoscale, vol. 10, no. 48, pp. 22745-22749, Dec 28 2018, https://10.1039/c8nr07854h. | 1 MW/cm^2^ |
| H. Jung, M. Lee, C. Han, Y. Park, K. S. Cho, and H. Jeon, "Efficient on-chip integration of a colloidal quantum dot photonic crystal band-edge laser with a coplanar waveguide", Opt Express, vol. 25, no. 26, pp. 32919-32930, Dec 25 2017, https://10.1364/Oe.25.032919. | 5 MW/cm^2^,  2.5 MW/cm^2^ |
| H. Chang et al., "Colloidal quantum dot lasers built on a passive two-dimensional photonic crystal backbone", Nanoscale, vol. 8, no. 12, pp. 6571-6576, 2016, https://10.1039/c5nr08544f. | 700 kW/cm^2^ |
| C. Dang et al., "Highly efficient, spatially coherent distributed feedback lasers from dense colloidal quantum dot films," Appl Phys Lett, vol. 103, no. 17, 2013, https://10.1063/1.4826147. | 900 kW/cm^2^ |
| This study | 10.5 kW/cm^2^ |

S6. Emission spectra from CQD-BIC laser structures prepared by drop casting and spin coating (without squeegee sweeping)


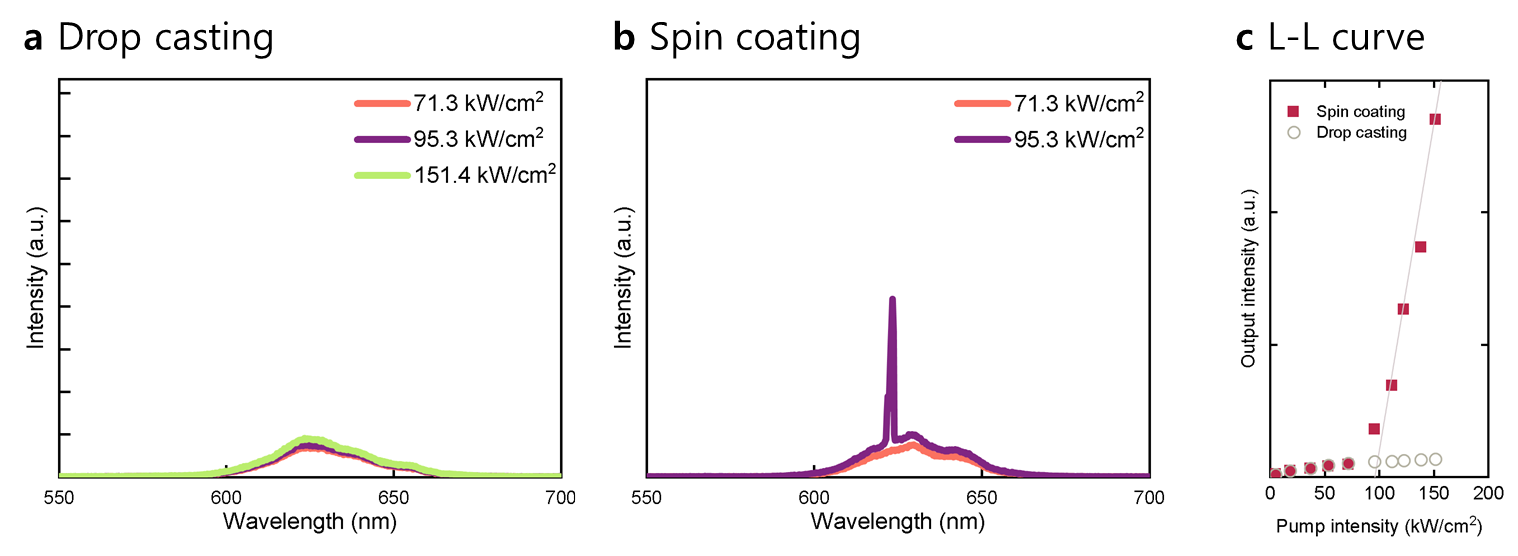


Fig. S6: Emission spectra of CQD-BIC structures with (a) drop-cast and (b) spin-coated films, both processed without squeegee sweeping. The drop-cast sample exhibits no lasing behavior, lacking discernible resonant modes due to the excessive amount of CQDs. In contrast, the spin-coated sample shows lasing with a threshold of 95.7 kW/cm²—approximately 10 times higher than the 10.5 kW/cm² threshold of the squeegee-swept CQD-BIC laser. (c) Output intensity versus pump intensity for the both samples.

S7. Complex refractive index dispersions of CQD film

**
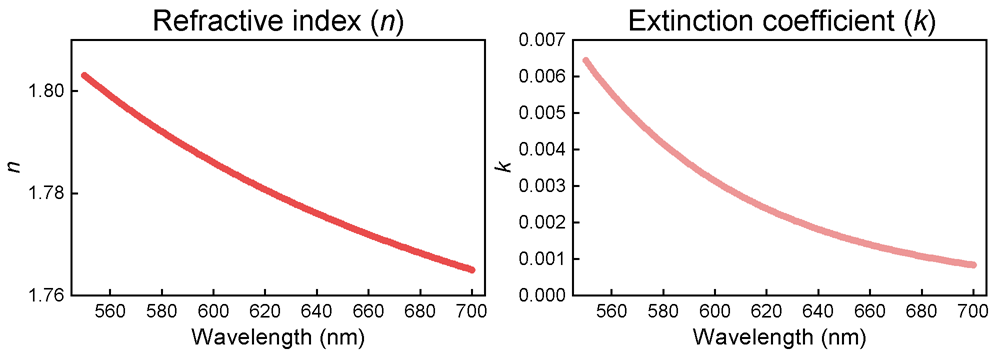
**

Fig. S7: Dispersions of the refractive index (*n*) and extinction coefficient (*k*) of the red CQD film used in the experiments, which were obtained from spectroscopic ellipsometry measurements.

S8. Photographic and optical microscopy images of the PDMS squeegee


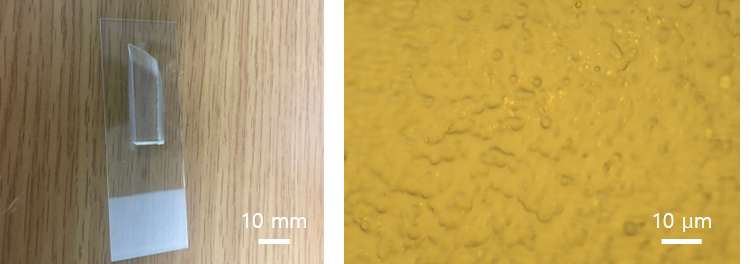


Fig. S8: Camera image (left) and optical microscopy image (right) of the PDMS squeegee on a microscope slide. The 1,000× magnified microscopy image reveals a smooth surface topology.

S9. CQD surface qualities without squeegee sweeping


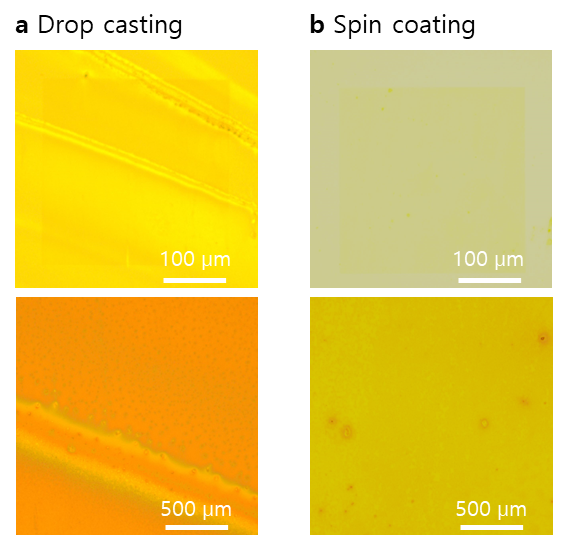


Fig. S9: Optical microscope images of CQD surfaces: (a) drop-cast and (b) spin-coated CQD films (no squeegee sweeping).
